# Alignment of histological and polarimetric large-scale imaging for brain tissue characterization

**DOI:** 10.1117/1.JBO.30.9.096003

**Published:** 2025-09-23

**Authors:** Éléa Gros, Omar Rodríguez-Núñez, Stefano Moriconi, Richard McKinley, Ekkehard Hewer, Théotim Lucas, Erik Vassella, Philippe Schucht, Tatiana Novikova, Christopher Hahne, Theoni Maragkou

**Affiliations:** a University of Bern, Institute of Tissue Medicine and Pathology, Bern, Switzerland; b University of Bern, Graduate School for Cellular and Biomedical Sciences, Bern, Switzerland; c Bern University Hospital, Inselspital, University of Bern, Department of Neurosurgery, Bern, Switzerland; d Bern University Hospital, University of Bern, University Institute of Diagnostic and Interventional Neuroradiology, Support Center for Advanced Neuroimaging (SCAN), Bern, Switzerland; e Lausanne University Hospital, University of Lausanne, Institute of Pathology, Lausanne, Switzerland; f IP Paris, Ecole Polytechnique, CNRS, LPICM, Palaiseau, France

**Keywords:** Mueller polarimetry, brain tumors, neuropathology, image processing, neuro-oncology

## Abstract

**Significance:**

Mueller polarimetric imaging shows great promise for differentiating neoplastic from healthy brain tissue during neurosurgery. However, validating algorithmic approaches is limited by the scarcity of substantial tumor border zones in *ex* *vivo* samples, limiting comprehensive analysis of tumor margins.

**Aim:**

We propose a protocol to build a database of histologically annotated polarimetric images from formalin-fixed whole-brain sections. We focus on validating the image alignment pipeline on healthy tissue.

**Approach:**

To address the size mismatch between samples and the field of view of imaging instruments, we developed an automatic reconstruction pipeline to create large-scale polarimetric images from smaller raster-scanned tiles. Matching points between reference photographs and tile images allowed precise alignment. Similarly, fractionated histological sections were reconstructed and accurately aligned with the polarimetric data to serve as ground truth.

**Results:**

The integrated reconstruction and alignment approach enabled large-scale, spatially co-registered polarimetric and histological imaging, supporting a more detailed investigation of tissue polarimetric parameters. The database thus created will facilitate the training and evaluation of segmentation models.

**Conclusions:**

The developed method improved polarimetry-based brain tissue mapping by linking polarimetric parameters with histological features, enhancing the quality and quantity of data available for training and evaluating segmentation models. Although initially applied to brain tissue, the protocol could be extended to other organs to support broader studies of polarimetric tissue characterization.

## 1 Introduction

### 1.1 Biomedical Applications of Mueller Polarimetry

Optical properties of biological tissues (e.g., depolarization, birefringence, and diattenuation) may be affected by pathological changes and serve as optical markers of a disease. Imaging Mueller polarimetry (IMP) is a technique proven useful for assessing these optical properties and revealing microstructural features of various healthy and neoplastic organ tissues.^1^^–^^3^ Polarimetry-based methods hold great promise to evaluate optical properties within tissues and characterize distinct layers of tissues in various organs, including colon,^4^ cervix,^5^ larynx,^6^ and brain.^7^ In addition, IMP presents several strengths for *in vivo* applications, including noncontact and contrast agent–free operation, the potential for real-time imaging,^8^ and the ability to capture wide-field images with a field of view (FoV) up to tens of square centimeters.

Prior studies have proven the effectiveness of polarized light imaging for the reconstruction of brain fiber tract organization.^9^^–^^11^ Furthermore, we have identified polarimetric biomarkers that enhance the image contrast between healthy and neoplastic brain tissue by accounting for randomization of the azimuth of the optical axis of brain white matter, which represents a linear birefringent medium.^7^ IMP has also demonstrated robustness to simulated neurosurgical environments.^12^^,^^13^ When combined with machine learning (ML) algorithms, IMP allows for differentiation between the white matter (WM), containing nerve fibers, and the surrounding gray matter (GM).^14^ Recent studies successfully employed IMP and ML for neoplastic tissue classification in other organs.^15^^–^^17^ With appropriate training data, advanced ML algorithms could enable automatic neoplastic brain tissue detection and delineation. If deployed during neurosurgery, a method showing a contrast between healthy and neoplastic brain tissue while visualizing fiber tracts would greatly enhance decision-making and improve patients’ prognosis by allowing more complete tumor resections in a safer manner.^18^^,^^19^

### 1.2 Challenges in Data Acquisition

In many organs of the human body, tumors are preferably resected *en bloc*, including the potentially infiltrated surrounding tissue, to avoid tumor recurrence. This is different in brain tumor surgery because the surrounding brain tissue harbors neurological function and is often key to the patient’s quality of life. Therefore, the goal of surgery is most often to remove the solid core of the tumor. As a result, virtually all fresh neoplastic brain tissue specimens available for research purposes do not contain healthy brain tissue. Hence, the border between healthy and neoplastic brain tissue is absent, complicating ML segmentation algorithm development and validation. While seeking to gather data from fresh brain specimens featuring both healthy and neoplastic brain tissue, an option consisted of measuring formalin-fixed whole-brain sections (WBS) collected following autopsies of brain tumor patients. These sections, which include both healthy and neoplastic brain tissue and the border between them, allow for a more thorough analysis of the polarimetric parameters by providing the context of the surrounding tissue, compared with smaller sections.

In our previous studies,^7^ we highlighted the importance of obtaining reliable ground truth (GT) maps in terms of histological characterization of brain tissue zones with a neuropathological diagnosis according to the World Health Organization classification for brain tumors.^20^ Working with formalin-fixed tissue would help collect accurate GT as fixing tissues typically helps to preserve tissue quality and morphology. Moreover, we have shown in a previous study that the effect of formalin fixation on the polarimetric properties of brain tissue is limited, and that fixed tissue may be used as a surrogate for fresh tissue.^21^

### 1.3 Multimodal Image Reconstruction and Alignment

**Fig. 1:**
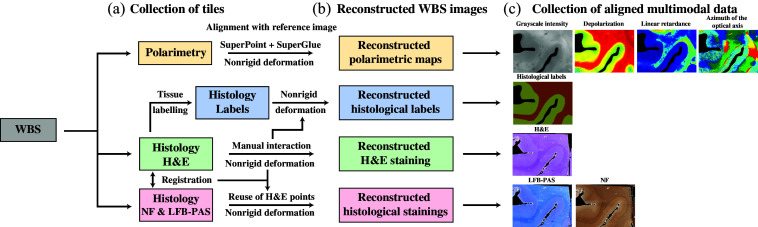
Workflow of the image acquisition and multimodal alignment pipeline. (a) Polarimetric tiles were acquired in a raster manner to cover the WBS before the block was cut into smaller patches for histological processing. (b) Polarimetric maps of the WBS were reconstructed automatically, whereas the H&E-stained histological images were reconstructed through a semi-manual alignment process. Histological labels were collected from the H&E images. Additional histological stainings were registered to the H&E images. The labels and stainings were then reconstructed using the nonrigid deformation computed for the H%E images. (c) This workflow enabled the collection of spatially aligned multimodal data, integrating polarimetric maps with multiple histological stainings.

Despite the potential of WBS, significant challenges remain. A major limitation is that the lateral dimensions of the WBS can be several times larger than the FoV of our IMP, making it impossible to directly image the entire section. This limitation is inherent to systems specifically designed for *in vivo* imaging. In contrast to transmission-mode systems, which are capable of imaging entire coronal WBS,^11^^,^^22^ our system is optimized for obtaining images from specific regions of interest during neurosurgical procedures, where real-time imaging and time efficiency are critical. In addition, the GT histological annotated images must also be aligned with the polarimetric images to enable accurate comparison between the two modalities and facilitate further investigation. Aligning neuroimaging data with histological images for validation is challenging due to tissue deformations introduced during histological processing, as well as differences in spatial resolution. Moreover, due to the tissue processing steps, histological data were acquired from physically disjoint tissue segments. This presents a significant challenge for large-scale image reconstruction and registration as most prior work assumes optically tiled acquisitions with spatial continuity and overlap.^23^^–^^25^ Blockface images and fiducial markers are commonly used as a tool to help estimate an optimal transformation when aligning MRI with histology.^26^ Although previous studies have explored alignment techniques for transmission-based polarized light imaging in controlled settings,^27^^,^^28^ these approaches are not directly applicable to reflection-mode systems designed for intraoperative use. To bridge this gap, we propose an image reconstruction and alignment pipeline tailored to formalin-fixed WBS and described in Fig. 1. This step is essential to establish a robust spatial framework for multimodal data integration and lays the groundwork for future applications involving annotation transfer and segmentation in samples with tumor margins. To this end, we present a comprehensive pipeline with three main steps, summarized as follows. First, we developed an automated method for polarimetric image reconstruction to overcome the FoV limitations inherent in our polarimetric imaging systems. The reconstructed polarimetric image was also aligned to a reference macroscopic WBS photograph (referred to as the “reference photograph”). Second, we established a semi-automatic protocol for reconstructing histological images of the WBS from a series of smaller histological tiles and aligning them to the same reference photograph. Third, we developed an automatic pipeline for reconstructing and aligning histological image tiles, regardless of the staining type, to the reference photograph, as long as the alignment has been performed for one type of staining. Using multiple histological staining images enables physicians to obtain comprehensive tissue diagnostics, enhancing the quality of their annotations.

It is worth noting that the reconstruction and alignment protocol is performed *ex vivo* and is not intended for real-time neurosurgical applications. Hence, there are no significant limitations regarding processing size, time, or other constraints in the *ex vivo* setup. Both histological and polarimetric images are aligned to the same reference photograph, ensuring high-fidelity image co-registration between modalities. This pipeline offers an enhanced dataset providing physicians and scientists the ability to link micro-structural changes in tissue with variations in polarimetric markers. Although applied here to healthy formalin-fixed tissue, the pipeline lays the groundwork for generating multimodal image datasets that include tumor borders, designed to be used for further training, testing, and validation of ML models. These models, once trained, can then be deployed for real-time segmentation of polarimetric images acquired intraoperatively using an IMP.

### 1.4 Our Contribution

This study presents the key steps of our pipeline, validated through the reconstruction and alignment of a WBS containing only healthy brain tissue. We first describe the sample and the procedure used to compare the performance of several local feature matching algorithms, applied to both polarimetric and histological images. After evaluating these algorithms, the most effective one was selected to identify and match keypoints between the polarimetric tile images and the reference photograph. These matched points were then used to align the polarimetric tiles with the reference image, thereby reconstructing large-scale polarimetric maps aligned to the reference photograph. The histological tiles were aligned to the same reference photograph, resulting in a satisfactory reconstruction and alignment of the histological image. We also used local feature matching algorithms on histological image pairs, finding matching keypoints, enabling the alignment of any histological staining once one kind was aligned. The resulting accurately aligned multimodal data provide diverse, high-quality GT, improving the robustness of tissue classification and segmentation models.

## 2 Methods

### 2.1 Imaging Mueller Polarimetry System

We examined a WBS using a wide-field, multiwavelength IMP system operating in reflection configuration within the visible wavelength range (450 to 700 nm), with an FoV of 2.5×2.1  cm, corresponding to an area of $5.25\;{\mathrm{cm}}^{2}$. Measurements were performed at 550 nm as the polarimetric system’s accuracy and detector sensitivity were optimized for this wavelength. This IMP system is well suited for biomedical tissue diagnosis, and its design, calibration, and optimization have been extensively discussed in previous studies.^10^^,^^12^^,^^13^^,^^21^^,^^29^^,^^30^ The system consists of an incoherent white light source, a polarization state generator to modulate the polarization of incident light, and a detection arm with a polarization state analyzer, spectral filter wheel, and charge-coupled device (CCD) camera for image acquisition. The polarization state generator includes a linear polarizer, two voltage-driven ferroelectric liquid crystals, and a waveplate placed between them. The polarization state analyzer mirrors the polarization state generator components in reverse order. Polarization modulation is performed sequentially.

To reconstruct the Mueller matrix, the system captures 16 intensity images by illuminating the sample with four linearly independent polarization states generated by the polarization state generator and projecting the scattered/reflected light into four linearly independent polarization states of the polarization state analyzer. Measurement precision is ensured by eigenvalue calibration.^31^ The signal-to-noise ratio is enhanced by repeating the set of measurements 8 times and averaging the results, along with applying a learning-based denoising framework.^8^ The recorded Mueller matrix images are processed using the Lu-Chipman polar decomposition algorithm,^32^ extracting maps of depolarization, linear retardance, azimuth of the optical axis, and grayscale reflected intensity. These parameters have been shown to be relevant for the optical diagnosis of brain tissue.^7^^,^^10^ For visualization, the azimuth of the optical axis was displayed in two formats: a full-resolution colormap and a quiver plot [Figs. 4(d) and 4(e)]. The quiver plot represents the averaged azimuth orientation within 25×25 pixel regions of interest, emphasizing large-scale patterns in birefringent structures. To ensure a clean and interpretable representation, regions with depolarization values below 0.88, mainly corresponding to GM, were masked from the quiver plot.

### 2.2 Sample Preparation and Processing

**Fig. 2:**
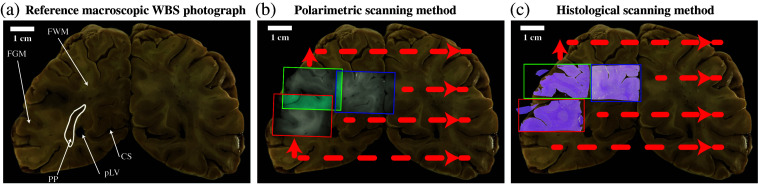
WBS collection and scanning process. (a) The reference photograph of the WBS, containing only healthy brain tissue and collected following an autopsy. FWM, forebrain white matter; FGM, forebrain gray matter; PP, posterior putamen; pLV, posterior horn of lateral ventricle; and CS, calcarine fissure. (b) Polarimetric image tiles (2.5  cm×2.1  cm) were acquired in a raster manner, with a significant overlap, to cover the entire surface of WBS. (c) The WBS was cut into 16 smaller patches for histological analysis: as the tissue is physically cut before staining, there is no overlap between the final histological image tiles.

A fixed WBS was collected following the autopsy of a patient without any brain dysfunction or disease [Fig. 2(a)], and thus the section only contains healthy brain tissue. The reference photograph was captured with a phone camera and had a resolution of 3650  pixels×2600  pixels. To ensure optimal image quality and limit photometric and geometric inconsistencies, the reference photograph was acquired with the camera placed at a normal angle to the sample surface under diffuse illumination, which reduces glare and shadows. The WBS was located in the parieto-occipital region of the brain. The lateral dimensions of WBS were 10×7  cm. This corresponds to a spatial resolution of about 30  μm/pixel. The total area of WBS was $\approx 70\;{\mathrm{cm}}^{2}$, making the FoV of the instrument ≈13 times smaller than the total area of WBS. The WBS had a thickness of 2 cm, which is 2 orders of magnitude larger than the penetration depth of polarized light in our settings (≈100μm^33^). Before measurements, the WBS was washed with distilled water and placed on a glass Petri dish.

We then acquired a series of polarimetric image tiles in a raster pattern [Fig. 2(b)], covering the entire WBS surface using the IMP system. Each tile measured ∼2.4×2.1  cm and was captured at a resolution of 516×388  pixels, corresponding to a spatial resolution of 50  μm/pixel. To facilitate the reconstruction of polarimetric images, we ensured a significant overlap of about 15% of the tile size between adjacent acquisitions [depicted in light blue, Fig. 2(b)]. Due to manual repositioning of the tissue section between measurements, the exact overlap value varied slightly, but remained close to 15%. A total of 15 measurements were performed to cover the entire WBS surface. For accurate GT on tissue pathological status, we collected histological data from the same WBS. After polarimetric measurements, the WBS was cut into small pieces for histological analysis [Fig. 2(c)], which were fit into standard histological cassettes (size of 2.8  cm×3.3  cm or $9\;{\mathrm{cm}}^{2}$). Overlap between histological tiles was not physically possible. To reduce variability in tissue processing and minimize errors during histology-to-polarimetry alignment, the tissue was fixed in 10% neutral buffered formalin for over 48 h, embedded in paraffin, then sectioned and stained by trained professionals using standardized protocols. A total of 16 tissue patches were stained with hematoxylin and eosin (H&E), Luxol Fast Blue combined with Periodic Acid-Schiff (LFB-PAS), and neurofilament, and the slides were digitally scanned. H&E provides details on cell structure, LFB-PAS stains myelin sheaths, and neurofilament highlights nerve fibers. The H&E slides were annotated by a certified board-certified neuropathologist using QuPath.^34^ For this study, the histological labels were limited to GM/WM, as only healthy brain tissue was present, but additional labels could be used for other samples, such as tumor cell infiltration levels.

The study was approved by the cantonal ethics committee of Bern (KEK BE 2019-02291) and follows the principles of the Declaration of Helsinki.

### 2.3 Local Feature Matching

Our pipeline for reconstructing and aligning histological and polarimetric images of WBS is based on local feature matching between image pairs to find sets of matching keypoints, which are used to constrain and regularize a nonlinear elastic deformation applied using bUnwarpJ.^35^ This method is based on B-spline deformation fields with vector spline regularization and includes a consistency term to model local tissue deformations (e.g., stretching or tearing) while ensuring symmetric alignment between the source and target images. Although manual keypoint selection was possible, we aimed to streamline the process and reduce user interaction by evaluating several automatic methods.

Local feature matching involves (1) detecting unique keypoints and generating corresponding descriptors, (2) using a nearest neighbor search to match keypoints across images, filtering out incorrect matches, and (3) optionally estimating a generic transformation. A common approach uses scale-invariant feature transform (SIFT)^36^ for keypoint detection and descriptor computation, followed by nearest neighbor-based matching. Incorrect matches are typically filtered using Lowe’s ratio test,^36^ mutual check, or neighborhood consensus strategies,^37^^,^^38^ and a transformation is estimated with RANdom SAmple Consensus.^39^ More recently, ML-based techniques have improved keypoint identification and descriptor computation, surpassing traditional methods such as SIFT.^40^^–^^43^ Additional information can also be obtained from models that offer broad yet coarse visual knowledge, aiding in determining the general similarity between keypoints and generalization to multiple domains.^44^ New outlier rejection techniques have also been developed to enhance match filtering,^45^^,^^46^ and attention-based graph neural networks have shown superior performance in specific tasks.^47^^,^^48^

We attempted to determine the combination of methods yielding the best local feature matches. Most models are trained using a set of images of the same modality. It, therefore, appears important to determine which methods perform the best with a set of images of different modalities. We compared the performance of methods using different keypoint detectors, namely SIFT,^36^ Simple Learned Keypoints (SiLK),^43^ Superpoint (SPoint),^42^ optionally coupled with DinoV2.^48^ Then, we fed keypoint matching algorithms, including mutual NN constraint (MNN), Order-Aware Network (OANet),^46^ Superglue (SGlue),^47^ and OmniGlue (OGlue).^48^ We used two databases, for two different tasks, to evaluate the performance of the different method combinations:

1. Reference polarimetry (RefPol) consists of 15 image pairs. Each pair includes the reference photograph (referred to as the “fixed image”) cropped to contain the region corresponding to the polarimetric tile and some surrounding area. The fixed image was aligned to the polarimetric tile (referred to as the “moving image”). The images in this database are of size 516×388  pixels.
2. Histology–Histology (${\mathrm{Hist}}^{2}$) consists of 16 image pairs. Each pair includes a grayscale version of the H&E staining, resized to a size of 1068×720  pixels (referred to as the “fixed image”). The fixed image was aligned with a version of itself deformed (referred to as the “moving image”), using a combination of a rigid transformation, used to represent different positions of the section on the histological slide, and an elastic deformation, used to represent local small deformations due to the sectioning step.

The output of each method consisted of the coordinates of matched landmarks between the fixed and moving images. For each match, we mapped the fixed point onto the moving image using a GT map. When aligning polarimetric tiles to the reference photograph, GT keypoints were manually selected and used to constrain an elastic deformation,^35^ generating a GT map. When aligning pairs of histological images, the GT map was derived by applying the known deformation to the original tile. We then computed the Euclidean distance between the GT point and the predicted point on the tile. This yielded a set of distances, from which we calculated the root mean square error (RMSE) to compare the performance of different method combinations

$$\mathrm{RMSE}=\sqrt{\frac{1}{n}\sum_{i=1}^{n}d_{i}^{2}},$$ (1)

where $d_{i}$ represents the Euclidean distance between the GT and predicted points and n is the number of distances. We also computed precision, defining true positives as points with a distance less than 15 pixels for RefPol (and less than 5 pixels for ${\mathrm{Hist}}^{2}$). False positives were defined as points with a distance greater than the respective threshold. These thresholds were chosen to account for small variations that do not significantly impact the precision of the deformation. The use of two thresholds reflects the distinct characteristics of the datasets: the 15-pixel threshold for RefPol accommodates its larger deformation scale, whereas the 5-pixel threshold for ${\mathrm{Hist}}^{2}$ ensures precision at its finer scale, required for histology images with a very high resolution.

### 2.4 Alignment Validation

A key control in this study was to validate the alignment of the reconstructed polarimetric and histological images with the reference photograph [Fig. 2(a)]. The validation pipeline relies on manual labels, and every manual interaction within the pipeline has been performed by trained researchers under the supervision of a senior neuropathologist. To assess the quality of the alignment, a GM/WM border mask was manually delineated on both the reference photograph and the reconstructed polarimetric map. These manually defined masks were used solely for validation purposes, more specifically to compute alignment metrics such as the dice score, and were not used to generate segmentation labels for training or analysis. The border mask for the reconstructed histological image was automatically extracted from the reconstructed labels, by selecting the pixels located at the border between GM and WM [Fig. 7(c)]. We then dilated the border line using an 11×11 kernel for five iterations and a 5×5 kernel for one iteration, resulting in a 55-pixel wide border line. Given that each pixel represents 0.029 mm in the reference photograph (and hence in the reconstructed polarimetric map and labels), it corresponds to a border line of 1.595 mm. This dilation coincided with the uncertainty region, which, based on previous studies, spans ∼1.59  mm.^21^ To quantitatively evaluate the overlap between the two border lines, we calculated the dice score

$$\mathrm{Dice}(X,Y)=\frac{2|X\cap Y|}{\left|X|+|Y\right|},$$ (2)

where X represents the border pixels from the reference photograph and Y represents the border line pixels from the reconstructed polarimetric or histological image.

To enhance the robustness of our alignment validation, we extended our analysis beyond the border between GM and WM, incorporating reference points distributed across the WBS. We manually selected a set of 55 reference points in the reference photograph, the reconstructed histological image, and the reconstructed polarimetric image. The points were selected in areas recognizable in the three modalities, including blood vessels, brain nuclei, and gyri. The alignment accuracy was then quantitatively evaluated using the RMSE between corresponding points across the modalities.

## 3 Experiments and Results

### 3.1 Alignment of Polarimetric Images to the Reference Photograph

#### 3.1.1 Alignment of polarimetric tiles

**Fig. 3:**
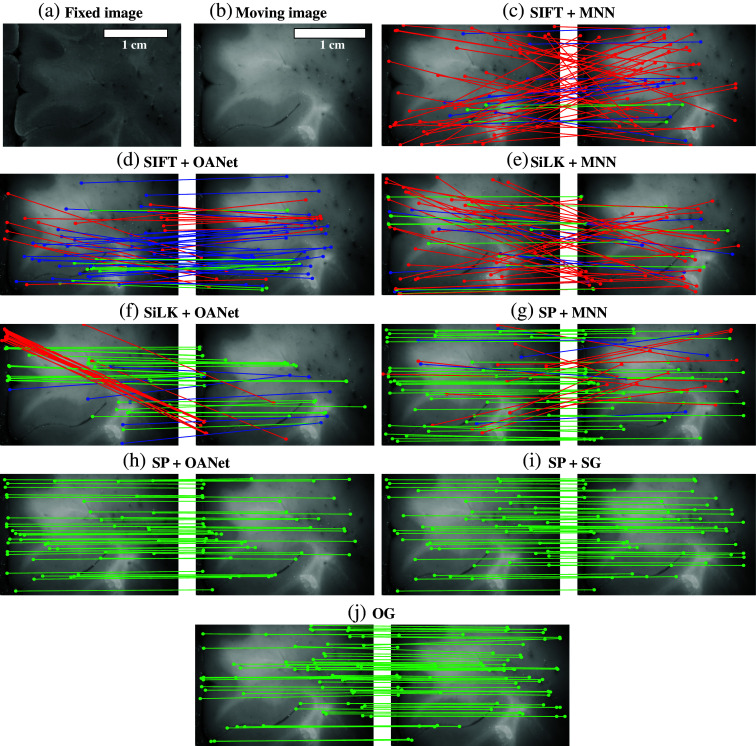
Qualitative local feature matches. Comparison of the position of the derived keypoints when matching the reference photograph (fixed image, a) and an example polarimetric tile image (moving image, b). Representation of the location of the points on the polarimetric tile returned by the different methods, on the left, and the corresponding GT on the right [(c)–(j)]. Match quality is color-coded: green indicates accurately matched keypoints (within the acceptable error threshold <15  pixels), blue denotes moderately accurate matches (within a tolerance margin >15 and <50  pixels), and red corresponds to incorrect or outlier matches (outside the error threshold >50  pixels).

In this first experiment, we reconstructed the WBS polarimetric maps and aligned them to the reference photograph. Here, the GT was generated using manually selected points, used to constrain and regularize an elastic deformation applied to the fixed image.^35^ This allowed us to generate a GT map between the pixels located in the fixed image and the moving image. We evaluated the performance of the different combinations of methods described in Sec. 2.3. Combinations of methods using SiLK for keypoint detection [Figs. 3(e) and 3(f)] outperformed the ones using SIFT [Figs. 3(c) and 3(d)] with an important improvement in the precision (3.60% SIFT + MNN versus 6.20% SiLK + MNN and 24.90% SIFT + OANet versus 43.00% SiLK + OANet, Table 1) and the RMSE value when combined with OANet (152.9 pixels SIFT + OANet versus 104.5 pixels SiLK + OANet, Table 1). However, there was no important improvement in the RMSE values when using MNN combined with SiLK compared with MNN combined with SIFT (Table 1). The combinations using SPoint for keypoint detection [Figs. 3(g)–3(i)] outperformed the ones using SIFT and SiLK for all matching methods. Both the RMSE value and the precision were improved significantly when using SPoint. OGlue outperforms all methods using SIFT and SiLK as keypoint detectors as well as the combination SPoint + MNN, improving greatly the RMSE and precision values [Fig. 3(j), Table 1]. However, OGlue is outperformed by two methods, which appear especially performant for such a task: SPoint + OANet and SPoint + SGlue. Both methods provide high precision (97.28% SPoint + OANet and 97.53% SPoint + SGlue, Table 1) and low RMSE values (6.6 pixels SPoint + OANet and 6.3 pixels SPoint + SGlue, Table 1). It is also worth noting that the performance of the methods using OANet for keypoint detection outperformed that of the ones using MNN for all keypoint detection methods [Figs. 3(c)–3(i), Table 1]. The combination of SPoint and SGlue outperformed all other methods [Fig. 3(h), Table 1] and was, therefore, chosen to extract matching keypoints.

**Table 1:** Performance of local feature matching algorithms applied to RefPol.

Method		RMSE (pixel)	RMSE (mm)	Precision (%)
SIFT	MNN	246.3±2.6	12.32±0.13	3.60±0.43
	OANet	152.9±5.8	7.64±0.29	24.90±1.81
SiLK	MNN	243.7±2.5	12.18±0.12	6.20±0.51
	OANet	104.5±2.7	5.22±0.13	43.00±0.93
SPoint	MNN	127.0±7.6	6.35±0.38	61.93±2.50
	OANet	6.6±0.5	0.33±0.02	97.28±1.15
	SGlue	6.3±0.5	0.32±0.03	97.53±1.23
OGlue		13.1±2.2	0.65±0.11	90.21±1.80

#### 3.1.2 Reconstruction of WBS polarimetric images

After aligning the polarimetric tiles with the reference photograph, we proceeded with the reconstruction of the WBS polarimetric maps. We implemented a method applying weights to the pixels, where the weight depended on the distance from the pixel to the closest pixel with no signal. This created a gradient effect that smoothed the transitions between the tiles. The parameter values for the full-scale polarimetric images were calculated as follows:

$${\bar{p}}_{g}=\frac{\sum_{p_{t}!=0}f_{t}(x,y)\times p_{t}}{\left\{p_{t}\neq 0\right\}},$$ (3)

where ${\bar{p}}_{g}$ is the parameter value for the reconstructed image and f is a weighting function for a pixel at location (x,y) in a given tile. The function f was defined as

$$f_{t}(x,y)=\frac{\mathrm{sig}(w(x,y))-\mathrm{sig}(0)}{\mathrm{sig}(1)-\mathrm{sig}(0)},$$ (4)

where the functions w and sig are defined as

$$w(x,y)=1-\frac{d_{\max}-d_{\mathrm{border}}(x,y)}{d_{\max}},$$ (5)

$$\mathrm{sig}(x)=\frac{1}{1+\exp(-\frac{2x-1}{0.2})},$$ (6)

where $d_{\max}$ is a user-defined parameter that sets the gradient length and $d_{\mathrm{border}}(x,y)$ is the distance from the pixel at location (x,y) to the nearest pixel with a value of 0 (i.e., located at the border) in the corresponding tile.

**Fig. 4:**
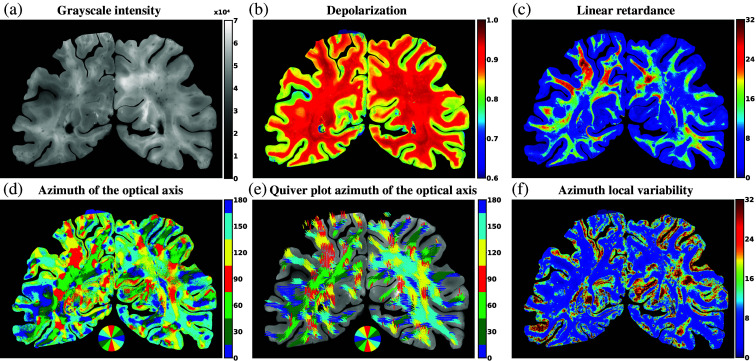
Reconstructed polarimetric maps. (a) Grayscale reflected intensity, (b) depolarization, (c) linear retardance, (d) azimuth of the optical axis (colormap representation), (e) quiver plot representation of the same azimuth of the optical axis data, and (f) azimuth local variability. The maps show expected contrasts between gray and white matter in healthy brain tissue.

The WBS-reconstructed polarimetric images are shown in Fig. 4. Small reconstruction artifacts, i.e., regions of the border of the tiles, are present in the grayscale intensity image [Fig. 4(a)]. The values of polarimetric parameters correspond to the typical values observed in brain tissue,^7^ showing a clear contrast between GM and WM in both the depolarization and linear retardance maps [Figs. 4(b) and 4(c)]. Furthermore, distinct fiber tracts running through the WBS hemispheres are visible in the WM regions of the azimuth of the optical axis maps [Figs. 4(d) and 4(e)], with low azimuth local variability values [Fig. 4(f)].

**Fig. 5:**
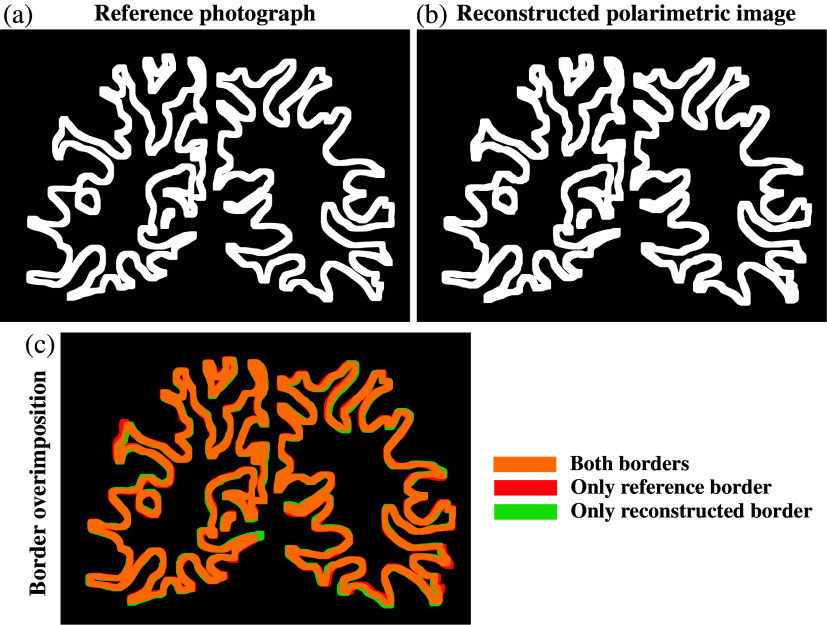
Validation of the alignment of the polarimetric images with the reference photograph. The reference photograph was used to generate manually (a) a mask delineating the GM and WM border. (b) The same border was defined with the same method using the reconstructed polarimetric maps. (c) An overlay of dilated border lines shows a close match between the two borders.

We then validated the alignment (see Sec. 2.4 for more details) of the reconstructed polarimetric image with the reference photograph. A manual mask was created to delineate the border region between GM and WM using both the reference photograph [Fig. 5(a)] and the aligned polarimetric images [Fig. 5(b)]. We overlaid the two images and found that the two border lines demonstrate a good overlap [Fig. 5(c)]. The resulting dice score of 0.91 further indicated a good alignment between the two borders.

### 3.2 Alignment of Histology Images to the Reference Photograph

#### 3.2.1 Alignment of histological tiles

For a second time, we aligned the images of scanned histological slides (hereafter referred to as “histological tiles”) with the reference photograph, hereby reconstructing the image of WBS histological staining. We collected a set of matching keypoints between the histological tiles and the reference photograph. However, to the best of our knowledge, no publicly available method has been developed for the specific application of co-registering fragmented histological images with wide-field photographs. The local feature matching algorithms discussed in Sec. 2.3 were all ineffective in generating useful information in this context, as the images are too dissimilar. Consequently, we opted to manually select matching points between the histological tiles and the reference photograph. More specifically, we selected keypoints that were easily recognizable in both the histological tiles and the reference photograph, such as the border between GM and WM, blood vessels, brain nuclei, and gyri.

Another important challenge in this alignment task is the very high resolution of the histological image and the need to be able to zoom in on the reconstructed image to acquire histopathological data. Despite their similar physical dimensions, a typical histological image can measure around 80,000×50,000  pixels, whereas a polarimetric image captured with our reflection-mode IMP has a much lower resolution, equal to 516×388  pixels. As the final resolution of the reconstructed WBS histological image needs to be several orders of magnitude larger than that of the reference photograph to capture essential details such as tissue morphology and cellular structure, the adjustments of the computing process were necessary. We computed the reconstructed WBS histological images in smaller subregions to reduce RAM usage and enable parallel processing. These computations were performed on a high-performance computing cluster, allowing for efficient handling of the large dataset sizes involved.

**Fig. 6:**
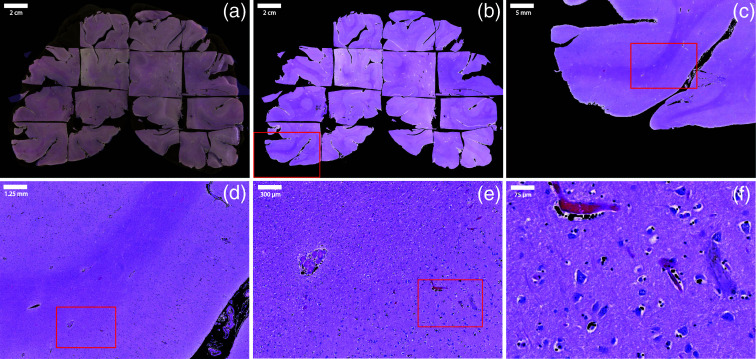
(a) Reconstructed H&E-stained histological image overlaid onto the reference photograph of the WBS. The histology overlay is shown with partial transparency to allow visualization of the underlying reference photograph. Some regions between the tiles are missing due to tissue processing limitations and the inability to create overlap between tiles; (b)–(f) sequentially zoomed-in views of the highlighted subregion (red rectangle) show sufficient resolution for a neuropathologist to detect the presence of tumor cells or any other abnormalities.

The reconstructed H&E-stained image is shown overlaid onto the reference photograph of the WBS in Fig. 6(a). Some regions between the tiles are missing, mostly due to tissue deformation during embedding and tissue loss during sectioning. Moreover, it is not physically possible to create overlapping areas between the histological tiles. A zoomed-in view of a specific subregion, highlighted by the red rectangle, demonstrates that the resolution is high enough for neuropathologists to extract valuable information from the staining on a single cell level [Figs. 6(b)–6(f)]. In addition, the reconstructed histological image is aligned with the reference photograph, and by extension, with the polarimetric image. This alignment enables visualization of the same region across both histological and polarimetric images, providing a comprehensive view for comparative analysis.

#### 3.2.2 Reconstruction of histological labels

**Fig. 7:**
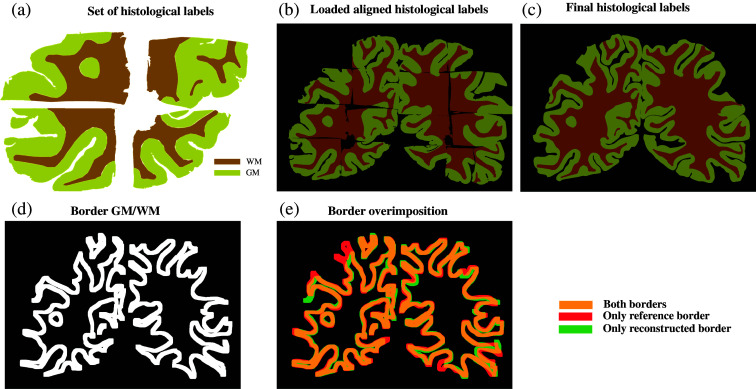
Reconstruction and validation of the histological label map. (a) Histological labels derived from H&E staining were extracted. (b) These labels were aligned to the reference photograph using the previously established map. (c) Missing regions in the histological label map were filled. (d) The border between GM and WM was derived from the reconstructed labels. (e) An overlay of the borders from the histological labels and the reference photograph reveals a general correspondence with minor discrepancies due to tissue sectioning during histological processing.

Aligning the histological tiles to the reference photograph also enabled us to reconstruct a histological label map. We extracted labels generated from the H&E-stained sections using QuPath [Fig. 7(a)]. Using the map obtained in the previous step, which maps the H&E staining on the reference photograph, we then aligned these histological labels to the reference photograph [Fig. 7(b)]. The histological labels were missing for some regions because of the gaps in the reconstructed WBS histology image. We filled these gaps using a custom-built algorithm that connects the borders of the WM regions in the different tiles, thus resulting in the reconstruction of the complete histological label map [Fig. 7(c)].

We further validated this reconstruction method by comparing the borders of the histological labels with the borders manually annotated from the reference photograph [Fig. 5(a)]. The reconstructed histological label map [Fig. 7(c)] was used to extract the border between GM and WM [Fig. 7(d)]. An overlay of the two borders [Fig. 7(e)] allowed us to assess their alignment. In comparison with the results of the same procedure for the polarimetric border, more discrepancies were observed between the reference and the histological borders. Some tissue image zones, especially on the sides of the WBS, were simply missing (visible impact on the middle to top left part of the image). Tissue processing steps, particularly the sectioning, remove several hundred micrometers of tissue and are potentially the reason for such discrepancies. As a result, the tissue being stained is not located exactly within the imaging plane of the reference photograph and polarimetric image (visible impact on the bottom to middle right part of the image). However, the borders generally coincide, with only minor deviations in some localized regions. The resulting dice score of 0.84 also indicates a satisfactory alignment between the two borders.

#### 3.2.3 Histological and polarimetric images alignment validation

**Fig. 8:**
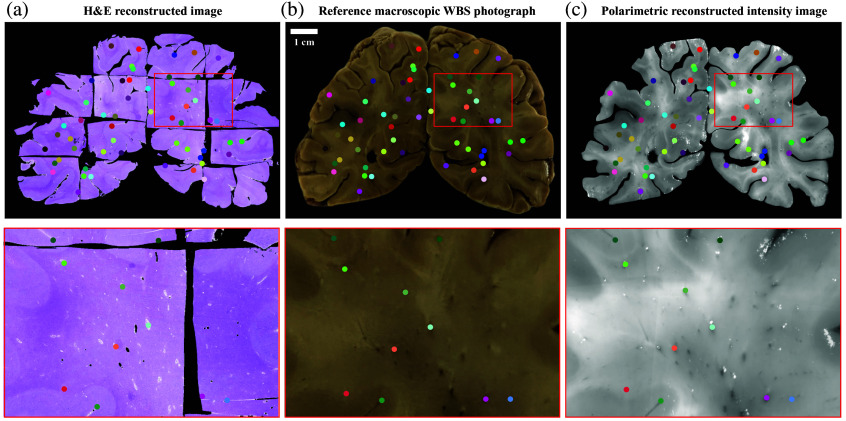
Validation of the alignment of the histological and polarimetric image.

Once the histological image and polarimetric image were reconstructed and aligned to the reference photograph, we aimed to validate the alignment between them (see Sec. 2.4 for more details). We selected matching points, distributed across the WBS in the reconstructed histological image, the reconstructed polarimetric image, and the reference photograph as a reference [Figs. 8(a)–8(c)]. The selected points are represented in a zoomed-up region, demonstrating a good alignment of the reconstructed images. We further quantified the RMSE of the selected points’ alignment between the histological and polarimetric images. The RMSE obtained of 7.96 pixels (corresponding to 226  μm) indicates an accurate alignment between the two modalities.

### 3.3 Alignment of Paired Histological Images

Although H&E staining provides valuable diagnostic information, we also aimed to perform additional staining, such as LFB-PAS and NF, and align the histology images to gain further diagnostic insights. To avoid the need for manually selecting matching points again, we “converted” the points selected for the H&E images into the reference photograph frame of the additional histological staining image. More specifically, we gathered a set of matching keypoints from the H&E-stained image and the other histological staining image. These keypoints were then used to guide the elastic deformation of the other histological staining image, allowing for mapping between the H&E and additional staining images. We were then able to transfer the points selected from the H&E image to the corresponding points in the additional histological staining images. We evaluated the performance of the different methods described in Sec. 2.3 using the database ${\mathrm{Hist}}^{2}$. The GT was generated in this experiment by mapping the points located on the original tile to the deformed tile by applying the deformation used to generate the deformed tile.

**Fig. 9:**
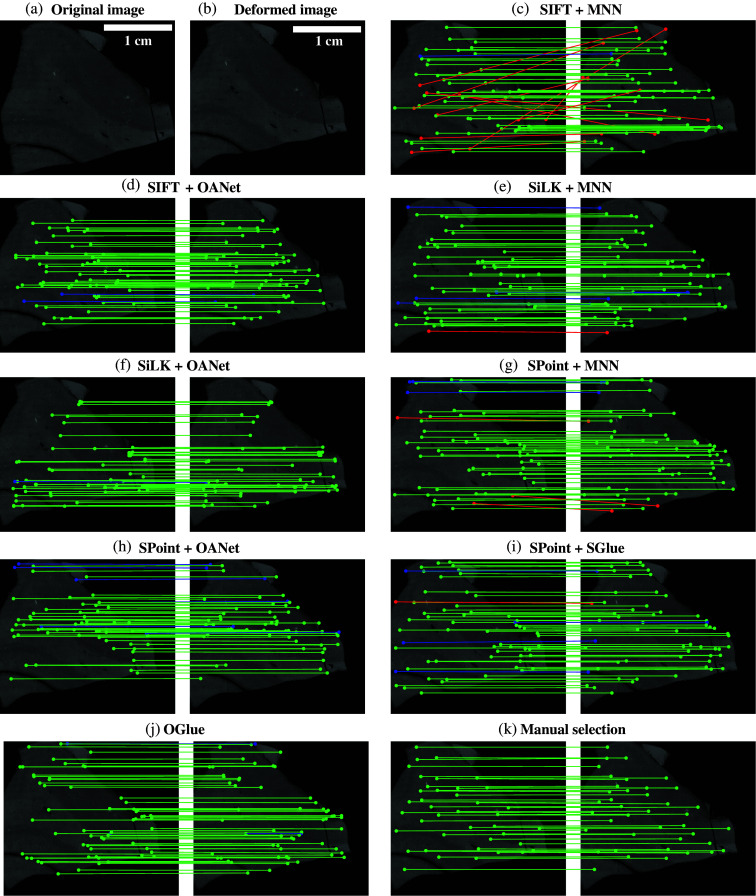
Qualitative local feature matches. Comparison of the position of the keypoints derived when matching (a) the original image and (b) the deformed image; (c)–(k) representation of the location of the points on the polarimetric tile returned by different methods (on the left) and the corresponding GT (on the right). Match quality is color-coded: green indicates accurately matched keypoints (within the acceptable error threshold <5 pixels), blue denotes moderately accurate matches (within a tolerance margin >5 and <10 pixels), and red corresponds to incorrect or outlier matches (outside the error threshold >10 pixels).

The combination of SIFT + MNN method greatly underperformed compared with the other methods, exhibiting a high RMSE value and relatively small precision value [Fig. 9(c), Table 2]. The remaining methods can be grouped into two distinct clusters based on their RMSE and precision values:

• Intermediate RMSE cluster: The methods SiLK + MNN, SiLK + OANet, SPoint + MNN, and SPoint + OANet form a cluster characterized by intermediate RMSE values ranging between 30 and 50 pixels. These methods also demonstrated relatively high precision, with values spanning from 93.70% (SPoint + OANet) to 98.25% (SiLK + OANet) [Figs. 7(e)–7(h), Table 2].
• Small RMSE cluster: The methods SIFT + OANet, SPoint + SGlue, and OGlue belong to a cluster producing smaller RMSE values, ranging from 1.57 (SIFT + OANet) to 3.21 pixels (SPoint + SGlue). Their precision values are similarly high, varying from 92.55% (SPoint + SGlue) to 98.87% (SIFT + OANet) [Figs. 9(d), 9(i), 9(j), Table 2].

**Table 2:** Performance of local feature matching algorithms for ${\mathrm{Hist}}^{2}$.

Method		RMSE (pixel)	RMSE (mm)	Precision (%)
SIFT	MNN	151.24±2.80	3.93±0.07	87.04±0.29
	OANet	1.57±0.12	0.041±0.003	98.87±0.11
SiLK	MNN	37.11±1.84	0.965±0.048	96.89±0.10
	OANet	49.92±3.29	1.297±0.085	98.25±0.12
SPoint	MNN	33.44±5.27	0.869±0.137	94.66±0.41
	OANet	37.81±10.79	0.983±0.280	93.70±0.67
	SGlue	3.21±0.15	0.083±0.004	92.55±0.46
OGlue		2.81±0.54	0.073±0.014	95.75±0.39
Manual selection		3.07±0.26	0.080±0.006	92.00±2.14

Although both clusters demonstrate comparable precision values, the second cluster achieved notably smaller RMSE values. A plausible explanation is that false positives in the first cluster are associated with larger point-to-point distances, which increases the RMSE values. An example of these “high-distance misses” is illustrated in Fig. 9(f). Based on its superior performance, SIFT + OANet was selected for this task, as it achieved the highest precision and the lowest RMSE. In addition, manually selected points by a user also exhibited an intermediate precision score (92.00%) and a low RMSE (3.07 pixels, Table 2), performing comparably to the SPoint + SGlue combination. Some errors in manual selection can likely be attributed to user bias of a few pixels, though this method avoids introducing “high-distance misses.”

**Fig. 10:**
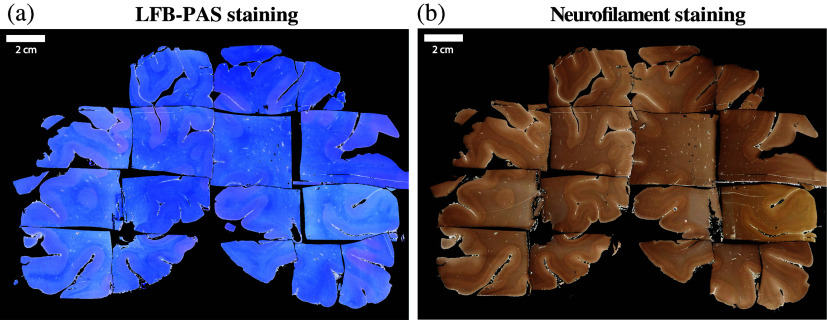
Results of the application of the reconstruction method developed for the H&E image to (a) LFB-PAS and (b) neurofilament staining images.

Finally, the method allowed us to successfully transform a set of points collected on the H&E image to additional histological staining images. The method was applied to the WBS for both LFB-PAS and neurofilament staining (Fig. 10), yielding a similar output in terms of the accuracy of reconstruction as for the H&E-stained histological image.

## 4 Discussion and Conclusion

This study presents a novel pipeline for reconstructing and aligning histological images with IMP data collected on WBS, enabling high-precision tissue characterization in large tissue sections. Our approach addresses challenges inherent to imaging large tissue sections, particularly those with lateral dimensions larger than the instrument’s FoV and the standard size of histological cassettes. Multitile image reconstruction and mosaicking are commonly applied to optically tiled datasets in which images are acquired from adjacent regions with physical continuity and overlap.^23^^–^^25^ Our approach tackles the more complex challenge of reconstructing physically segmented tiles as the histological preparation involves cutting and processing spatially disjoint pieces of tissue, resulting in high-resolution slides with no physical overlap. Moreover, the images from polarimetry and histology differ vastly in contrast, resolution, and modality. To bridge this gap, we propose a pipeline that reconstructs and aligns WBS images from both modalities to a shared macroscopic reference photograph. This is done automatically for the polarimetric images and semi-automatically for the histological images, ensuring high spatial accuracy across modalities. We adapted a strategy originally explored in MRI-to-histology workflows^27^ to the context of optical imaging.

A key aspect of this pipeline is the integration of local feature matching methods across different imaging modalities. Such methods are typically trained using a set of images of the same modality, altered using a combination of, among others, photometric distortion, rotation, and capture of the image from different points of view, but they are usually not tested with images of different modalities. The combination of SPoint and SGlue algorithms proved to be highly effective, achieving an RMSE value of 6.3 pixels (0.33  mm) and a precision score of 97.53% when aligning polarimetric tiles to the reference photograph. Nevertheless, alignment accuracy was limited by method performance for some modalities, especially with lower precision approaches such as SIFT and MNN. This was not the case when aligning pairs of histological images as both images shared the same modality and were very similar. Previous studies have addressed the co-registration of different whole-slide histological images, typically to visualize the same tissue either as a composite 2D map or as part of a 3D reconstruction from serial sections. ^49^^–^^51^ By contrast, our pipeline uses this intra-histology alignment as a bridge to enable the integration of high-resolution histological images with polarimetric data. Moreover, future improvements may benefit from leveraging deep learning–based matching techniques tailored to cross-modal image data or incorporating transformations to better standardize across modalities.^52^^–^^54^

The ability to create accurately aligned, high-resolution histological and polarimetric datasets is critical for advancing ML in tissue segmentation. The robust alignment process is essential for downstream applications, including ML algorithm training, whereas accurate correspondence between imaging modalities is paramount for model reliability. Although our current study compares registration methods on a single specimen, we note that intrinsic variability (e.g., across GM and WM regions) and standardized protocols mitigate potential biases. Future validation on larger datasets will further strengthen these findings. This pipeline’s multimodal approach provides the means to build an enriched dataset that could improve ML model accuracy in detecting and delineating healthy and neoplastic brain tissue. Notably, having accurately aligned histological and polarimetric images enables more reliable model training by providing diverse, high-quality GT data, thus improving the robustness of tissue classification and segmentation algorithms. This study focuses on aligning *ex vivo* samples, with the primary objective of training segmentation models on a high-quality multimodal dataset. By leveraging a robust training dataset, we aim to enhance the reliability of polarimetry-based neurosurgical guidance without requiring histological processing during surgery. Although these models are not yet intended for intraoperative use, the *ex vivo* phase represents a crucial step toward their future deployment in real-time surgical settings.

**Fig. 11:**
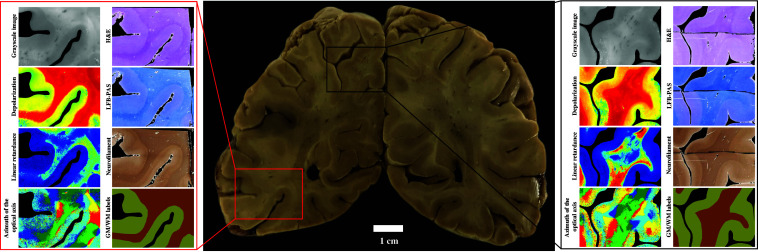
Set of the aligned multimodal data from two specific regions of interest shown by the black and red boxes in the WBS reference photograph. For each region of interest, the polarimetric maps are shown in the left column, and the histological staining and labeled images are shown in the right column.

Moreover, accurate alignment of multiple histological staining (e.g., H&E, LFB-PAS, and neurofilament) images provides physicians and scientists with comprehensive tissue diagnostics. Histological staining may help identify key tissue regions, such as tumor margins and transition zones between healthy and neoplastic brain tissue, for ML model training and validation of the model predictions. Moreover, they help address potential misclassifications and investigate causes in tissue microstructure, ensuring that the segmentation model is more accurate and reliable when applied to real-time clinical settings. Such advancements could lead to better differentiation between tissue types based on histological characteristics and polarimetric biomarkers, ultimately enhancing ML-driven tumor detection and boundary delineation in medical imaging. Scientists and physicians can navigate across different modalities, facilitating a comprehensive multimodal analysis of tissue properties (Fig. 11, online visualizer available here). One might, therefore, understand how and why the microstructural changes lead to the changes in the observed polarimetric images. We believe that such multimodal databases would allow us to better understand the disease phenotypes and, ultimately, to improve patient care.

Although designed for brain tissue, the proposed framework might be adapted for application to other complex organ tissues. The presented approach, particularly with the focus on spatially accurate image reconstruction and alignment of multimodal data, could be extended to additional biomedical imaging applications requiring precise microstructural information. One potential improvement is validating alignment methods across multiple specimens to account for biological variability. In addition, we could enhance the robustness of the reconstruction algorithm by implementing adaptive alignment metrics that adjust based on tissue type or imaging conditions. Automating manual labeling through a dedicated ML model could further improve efficiency.

In summary, the proposed alignment pipeline facilitates precise multimodal imaging. By providing a framework that aligns and reconstructs the images of large, complex tissue samples across histological and polarimetric modalities, we strongly believe that this work contributes to advancing polarimetric imaging’s clinical applicability and reliability. Future research should focus on refining image matching algorithms and exploring broader applications within and beyond neuro-oncology, where accurate tissue characterization remains critical.

## Data Availability

The code used for the comparison of matching methods, as well as the datasets RefPol and $Hist^{2}$, are publicly available at https://github.com/eleagros/clfm.
